# Genome evolution in trypanosomatid parasites

**DOI:** 10.1017/S0031182014000894

**Published:** 2014-07-28

**Authors:** ANDREW P. JACKSON

**Affiliations:** Department of Infection Biology, Institute of Infection and Global Health, University of Liverpool, Liverpool Science Park Ic2, 146 Brownlow Hill, Liverpool L3 5RF, UK

**Keywords:** *Trypanosoma*, *Leishmania*, genome, evolution, phylogeny, parasitism

## Abstract

A decade of genome sequencing has transformed our understanding of how trypanosomatid parasites have evolved and provided fresh impetus to explaining the origins of parasitism in the Kinetoplastida. In this review, I will consider the many ways in which genome sequences have influenced our view of genomic reduction in trypanosomatids; how species-specific genes, and the genomic domains they occupy, have illuminated the innovations in trypanosomatid genomes; and how comparative genomics has exposed the molecular mechanisms responsible for innovation and adaptation to a parasitic lifestyle.

## INTRODUCTION

Trypanosomatids are unicellular flagellates and obligate parasites that infect various animals and plants. They include *Trypanosoma* and *Leishmania*, species of which cause potent vector-borne diseases in humans, livestock and wildlife; diseases that are responsible for substantial mortality and morbidity across the world. *Trypanosoma cruzi* causes Chagas disease in South and Central America; *Trypanosoma brucei* causes Human African Trypanosomiasis in sub-Saharan Africa (and, along with related species, a similar disease in livestock); while *Leishmania* spp. cause various forms of leishmaniasis in humans. Other species of *Trypanosoma* and *Leishmania* infect a wide range of vertebrate hosts, and all are transmitted by invertebrate vectors; predominantly these are biting insects, although some aquatic species are transmitted by leeches (Lom, 1979). *Phytomonas* spp. are plant parasites transmitted by phloem-sucking insects and are occasionally an agricultural problem in South and Central America (Camargo, 1999). Besides these dixenic (i.e. two-host) parasites that cycle between insect/leech and vertebrate/plant hosts, the trypanosomatids include various other genera, such as *Crithidia, Leptomonas, Herpetomonas, Angomonas* and *Strigomonas* that are cosmopolitan, monoxenic (i.e. one host) parasites of insects (Maslov *et al.* 2013). The diverse associations of trypanosomatids indicate that the origin of parasitism is singular and ancient (Simpson *et al.* 2006).

**Fig. 1.**
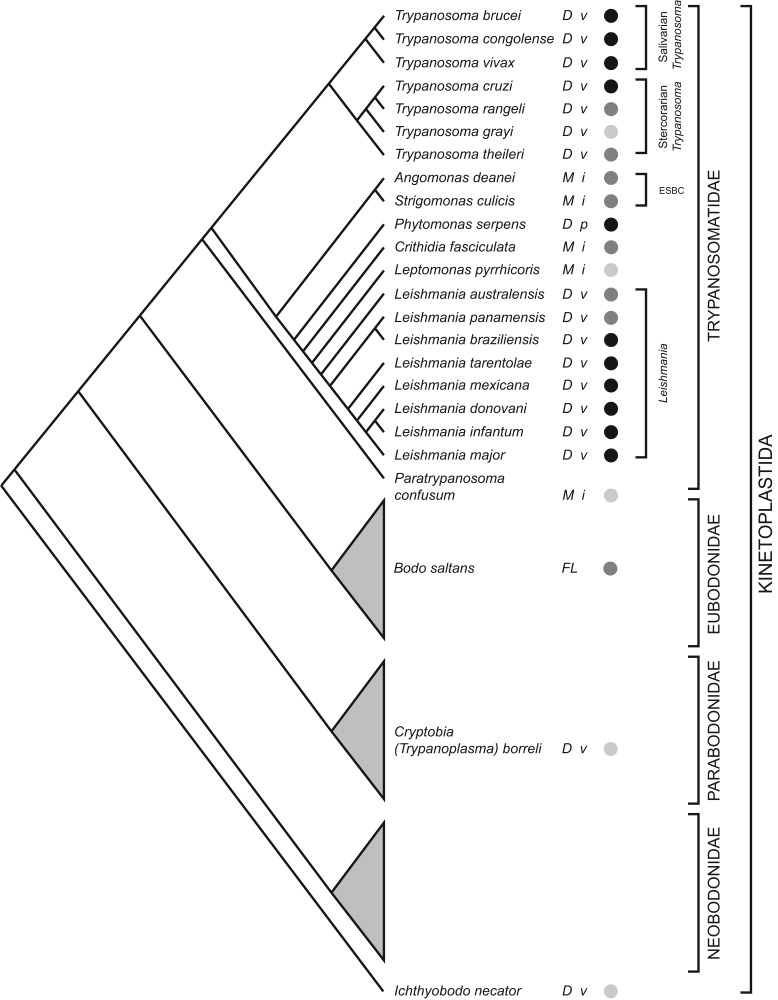
Kinetoplastid phylogeny. A cladogram depicting the current consensus on Kinetoplastid phylogenetic relationships (adapted from von der Heyden *et al.* 2004; Simpson *et al.* 2004, 2006; Deschamps *et al.* 2011; Flegontov *et al.* 2013). Each bodonid order is shown as a grey triangle, representing an indeterminate, but large, number of species. The status of genome sequencing projects for each named species is indicated by filled circles (black: finished; dark grey: unfinished draft; light grey: sequencing in progress). The life cycle of each species is indicated (*D*: dixenic; *M*: monoxenic; *FL*: free-living), as well as the host type(s) (*v*: vertebrate; *i*: insect; *p*: plant). ESBC: ‘Endosymbiont-bearing clade’.

The order Trypanosomatidae is one part of the phylum Kinetoplastida; most other Kinetoplastids live freely or as commensals in marine, terrestrial and aquatic environments. The current consensus on Kinetoplastid phylogeny is summarized in Fig. 1; trypanosomatids are monophyletic and the sister clade to eubodonids (Callahan *et al.* 2002; Simpson *et al.* 2004; Moreira *et al.* 2004; von der Heyden *et al.* 2004; Deschamps *et al.* 2011). The closest known relative among eubodonids is *Bodo saltans*, a free-living bacteriovore of terrestrial and freshwater microbiota. Hence, the phylogeny indicates that parasitism in trypanosomatids had a single origin; although the position of the fish parasites *Cryptobia* spp. and *Ichthyobodo* spp. show that parasitism has appeared on other occasions within the Kinetoplastida (Simpson *et al*. 2006; von der Heyden *et al.* 2004). This is the context in which I review the contribution of trypanosomatid genome sequences to our understanding of how parasitism evolved and subsequently diversified.

Since the publication of the ‘TriTryp’ genome sequences for *T. cruzi, T. brucei* and *Leishmania major* in 2005 (Berriman *et al.* 2005; El-Sayed *et al.* 2005*a*; Ivens *et al.* 2005), there has been much comparative analysis of these seminal resources. They have been complemented by transcriptomic (Holzer *et al.* 2006; Leifso *et al.* 2007; Saxena *et al.* 2007; Rochette *et al.* 2008, 2009; Alcolea *et al.* 2009, 2010; Depledge *et al.* 2009; Jensen *et al.* 2009; Kabani *et al.* 2009; Minning *et al.* 2009; Veitch *et al.* 2010; Adaui *et al.* 2011) and proteomic analyses (Atwood *et al.* 2005; Rosenzweig *et al.* 2008*a*, *b*; Alcolea *et al.* 2011; Eyford *et al.* 2011; Urbaniak *et al.* 2012; Butter *et al.* 2013) of gene expression at various life-cycle stages. Genome sequences for additional species of *Trypanosoma* (Jackson *et al.* 2009, 2012), *Leishmania* (Peacock *et al.* 2007; Downing *et al.* 2011; Rogers *et al*. 2011; Raymond *et al.* 2012; Real *et al.* 2013) and *Phytomonas* (Porcel *et al.* 2014) have been produced, with several more in progress (see Fig. 1).

Comparison of the Tritryp genomes showed that both gene order and gene repertoire are broadly conserved within chromosomal cores (El-Sayed *et al.* 2005*b*). It is generally thought that the considerable co-linearity displayed by trypanosomatid genomes, despite their apparently ancient divergences, reflects strong and fundamental selective constraints on genome structure (Ghedin *et al*. 2004). Analysis of gene order conservation across Eukaryotic genomes indicates that highly conserved gene pairs are retained for both functional and transcriptional regulation (Dávila-López *et al*. 2010). While there is little to suggest that the conserved proximity of genes in trypanosomatids reflects their shared or related functions, it has been suggested that their polycistronic organization necessitates the co-directionality of replication and transcription (Ghedin *et al.* 2004), and that this structural peculiarity of trypanosomatids (the cause of which remains unsolved), is responsible for the strong purifying selection that maintains gene order.

Beyond the chromosomal cores, within sub-telomeric regions for instance, there are numerous species-specific features (El-Sayed *et al.* 2005*b*). From the outset it was appreciated that these genes are very often associated with disease mechanisms (El-Sayed *et al.* 2005*b*) and are the basis for the distinctive cell surface architectures displayed by each parasite (Acosta-Serrano *et al.* 2007; Handman *et al.* 2008). Thus, after 10 years of comparative and experimental analysis of these genomes the principal genomic features that distinguish the stem trypanosomatid lineages, and which are most likely to have been instrumental in the evolution of parasitism, are apparent.

## GENOMIC REDUCTION

Parasites were once thought to be ‘degenerate’; while this view is no longer prevalent, it remains intuitive that some characters vital to free-living organisms, but no longer necessary for parasites within a host environment, are lost when the selection pressure to retain them is removed. Hence, we expect phenotypic reduction, which is often observed of parasites, to be reflected in genomic reduction. For example, the genomes of both schistosomes and cestodes, which are phenotypically reduced relative to free-living platyhelminthes, lack elements of canonical metazoan metabolism and developmental regulation (Berriman *et al.* 2009; Tsai *et al.* 2013). Genome reduction reaches its apogee in the microsporidian parasites, which in some cases have reduced their genomes to the physiological minimum required for life, and this corresponds with their extreme host dependence (Nakjang *et al.* 2013). At such extremes, we also observe physical compaction of the genome, in addition to the loss of genes (Keeling and Slamovits, 2005).

Trypanosomatids do not appear to be reduced physically; the size of their genomes (25–35 mb in the haploid state) and the gene density (2·8–4·6 Kb/gene) is comparable with free-living unicellular eukaryotes, for instance *Saccharomyces cerevisiae* (12·5 mb/2·09 Kb/gene) and *Dictyostelium discoideum* (33·8 mb/2·72 Kb/gene). However, trypanosomatid genomes might still be functionally reduced, having lost genes essential to free-living Kinetoplastids.

Before the advent of genome sequences, it was known that trypanosomatids lacked certain common metabolic capabilities. For example, they are auxotrophic for pteridine and folate, which are essential co-factors in macromolecule biosynthesis, because they lack the ability to synthesize tetrahydrobiopterin (Beck and Ullman, 1990, Bello *et al.* 1994, Nare *et al.* 1997; Ouellette *et al.* 2002). Similarly, they must scavenge haem from their hosts (or obtain it from bacterial endosymbionts; Alves *et al.* 2011), because they lack a native haem biosynthesis pathway (Chang *et al.* 1975; Korený *et al*. 2010). Trypanosomatids are also auxotrophic for purines (Marr *et al.* 1978; Gutteridge and Gaborak, 1979), vital in the biosynthesis of nucleic acids and energy metabolism. Other aspects of model eukaryotic physiology are also absent, for example, a system of redox homoeostasis based on catalase and glutathione reductase. Instead, trypanosomatids rely on a unique thiol-based redox metabolism based on trypanothione for the deactivation of oxidizing agents (Oza *et al.* 2005; Krauth-Siegel and Comini, 2008; Comini and Flohé, 2013). The initial Tritryp comparison showed that trypanosomatids do not possess receptor-linked tyrosine kinases (Parsons *et al.* 2005), canonical mitochondrial import systems (Pusnik *et al.* 2009), known telomere end binding proteins such as POT1 (Lira *et al.* 2007), certain genes that regulate autophagy (Herman *et al.* 2006) and others controlling apoptosis (i.e. TNF-related family receptors, Bcl-2 family members and caspases; Smirlis *et al.* 2010).

The question relating to these and any other missing features is whether they represent evolutionary losses, or instead, reflect the branching position of the Kinetoplastida in the eukaryotic phylogeny. It may be that certain widely conserved genes are absent from trypanosomatids because Kinetoplastids separated from other eukaryotic lineages early in evolutionary history and before those genes evolved. Furthermore, it could be that we have systematically underestimated genomic and physiological diversity in eukaryotes, and the apparent deficiencies of trypanosomatids reflect a biased perception based on a narrow sampling of animal and plant genomes. In short, the absence of ‘typical’ features from trypanosomatids need not represent evolutionary loss. In fact, detailed comparisons in the years following publication of the Tritryp genome sequences showed that, while trypanosomatids often lack some conserved features and have numerous clade-specific derivations, they are nevertheless comparable to free-living protists in the number and diversity of protein kinases (Parsons *et al.* 2005; Bahia *et al.* 2009), phosphatases (Brenchley *et al.* 2007), GTPases and other genes involved in intracellular trafficking (Field, 2005; Field *et al.* 2007) and DNA helicases (Gargantini *et al.* 2012).

In summary, these genomes are not reduced in size or substantially reduced in function. While trypanosomatids employ unique solutions in redox homoeostasis, mitochondrial protein import and telomere regulation, they nonetheless have a broadly typical eukaryotic physiology. Where there are disparities, it is not clear whether these genes were lost or never existed and this will only become clear after we have sampled the genomes of free-living Kinetoplastids for comparison. Instead, there is abundant evidence that trypanosomatid genomes have expanded during their evolution both physically, through the evolution of sub-telomeres and accessory chromosomes, and functionally, with the acquisition of new genes through duplication and horizontal gene transfer.

## GENOMIC INNOVATION: SPECIES-SPECIFIC GENE FAMILIES

Trypanosomatid cell surfaces include various polymorphic proteins combined with diverse glycolipid conjugates (Ferguson, 1997). These structures are enigmatic and their origins are mysterious because they are not seen in other organisms; indeed, the highly abundant cell-surface glycoproteins of *T. brucei, T. cruzi* and *L. major* are mutually exclusive, making it very hard to infer what the ancestral cell surface looked like (El-Sayed *et al.* 2005*b*). The Tritryp genomes revealed the genes that encode these surface features and their non-random distribution in the genome, which has been reviewed in detail elsewhere (Acosta-Serrano *et al.* 2007; Handman *et al.* 2008; De Pablos and Osuna, 2012). These cell surface proteins attract considerable interest because they are implicated in disease, virulence and mechanisms of pathogenesis (De Pablos and Osuna, 2012). Species-specific genes provide the clearest insight into genomic innovations associated with parasitism and the multi-copy gene families that encode these cell surface proteins dominate such species-specific genes in comparative analyses (El-Sayed *et al.* 2005*b*).

**Fig. 2.**
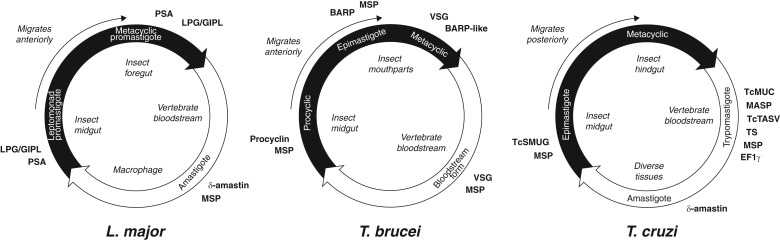
Trypanosomatid life cycles. Each circle represents the movement of parasites between insect (black) and vertebrate (white) hosts, showing the transition of parasite life stages, the position of each stage within the hosts and the timing of expression of cell surface-expressed protein families mentioned in the text. Note that for *T. brucei* and *L. major*, the parasites move anteriorly from the insect gut as they develop, while *T. cruzi* migrates posteriorly as it prepares for transmission into the vertebrate.

The life cycles of the Tritryp species and the points at which species-specific cell surface proteins are expressed are shown in Fig. 2. Species-specific genes in *T. cruzi* are dominated by gene families that encode the mucin-based surface coat during its trypomastigote stage (Cerqueira *et al*. 2008; Nakayasu *et al.* 2009; De Pablos and Osuna, 2012); primarily mucins (TcMUC; Acosta-Serrano *et al.* 2001; Buscaglia *et al.* 2006) and trans-sialidases (TS; Kim *et al.* 2005; Montagna *et al.* 2006; Freitas *et al.* 2011; Oppezzo *et al*. 2011; Ammar *et al.* 2013; Oliveira *et al.* 2014), but also a ‘dispersed gene family’ (DGF-1; El-Sayed *et al.* 2005*b*; Kawashita *et al.* 2009; Lander *et al.* 2010), the mucin-associated surface protein family (MASP; El-Sayed *et al.* 2005*b*; Bartholomeu *et al.* 2009; dos Santos *et al.* 2012), and the *T. cruzi* Trypomastigote Alanine, Serine and Valine-rich proteins (TcTASV; García *et al*. 2010; Bernabó *et al*. 2013). Gene families specifically expressed in the other life stages include amastin in the intracellular amastigote stage, and *T. cruzi* Small MUcin-like Genes (TcSMUG; Urban *et al.* 2011) in the replicative epimastigote. In addition to these developmentally regulated, surface-expressed gene families, expansions of Retrotransposon Hotspot (RHS) genes and Elongation Factor 1 gamma (EF1*γ*) genes are prominent innovations of the *T. cruzi* genome.

In *T. brucei*, species-specific genes are dominated by those encoding the Variant Surface Glycoproteins (VSG) that form the surface glycocalyx of all salivarian trypanosomes during their bloodstream stage in the mammal host (Hutchinson *et al.* 2007; Marcello and Barry, 2007; Jackson *et al.* 2012; Weirather *et al.* 2012; Hall *et al.* 2013). Other species-specific genes like the Invariant Surface Glycoprotein (ISG) genes (Jackson *et al*. 1993; Ziegelbauer and Overath, 1993) and Expression-Site Associated Genes (ESAGs; Pays *et al.* 2001; see below) are also preferentially expressed in the bloodstream stage. In the insect host, species-specific genes are dominated by procyclin, encoding the major surface glycoprotein of the procyclic stage while in the insect midgut (Roditi *et al.* 1998; Berriman *et al.* 2005), and the Brucei Alanine-Rich Protein (BARP) that, along with related forms, is specifically expressed by the epimastigote and metacyclic stages while in the insect mouthparts (Urwyler *et al.* 2007; Jackson *et al.* 2013).

The cell surface of *Leishmania* is dominated by non-protein lipophosphoglycan (LPG) and glycoinositolphospholipid (GIPL) molecules (de Assis *et al*. 2012). The LPG/GIPL coat is complemented by species-specific, multi-copy proteins such as *δ*-amastin, which is specifically expressed during the intracellular amastigote stage (Rochette *et al.* 2005). While its function is unknown, the evolution of *δ*-amastin is thought to be an adaptation for infection of, or survival within, macrophages since it is absent from monoxenic species (*Crithidia* and *Leptomonas* spp.) lacking a vertebrate stage (Jackson, 2010) and less abundant in *Leishmania* species that do not routinely infect macrophages (Raymond *et al.* 2012). Furthermore, a parallel expansion of *δ*-amastin has occurred in *T. cruzi*, which also has an amastigote stage, and this is associated with virulence (Kangussu-Marcolino *et al.* 2013). Another *Leishmania*-specific family, tuzin (Ivens *et al.* 2005), is linked to *δ*-amastin loci physically and phylogenetically (Jackson, 2010); hence, tuzin might be involved in the same adaptation. In the insect life stage, the promastigote surface antigen (PSA or gp46) is preferentially expressed in metacyclic promastigotes (Handman *et al.* 1995) and is encoded by a diverse gene family in human-infecting species (Devault and Bañuls, 2008). Also specifically expressed in metacyclics are the HASP (Hydrophilic Acylated Surface Protein) and SHERP (Small Hydrophilic ER-associated Protein) gene families (Depledge *et al.* 2010; Sádlová *et al*. 2010).

While the precise functions of these enigmatic gene families are unknown, several contribute to parasite fitness. This may be because they initiate infection, for instance, the TcMUC and TS proteins interact to transfer host sialic acid residues to parasite mucins, which is essential for attachment and invasion by *T. cruzi* trypomastigotes (Acosta-Serrano *et al.* 2001; Oliveira *et al.* 2014). Other cell surface protein families are essential for parasite development and transmission through the insect host; for example, HASP and SHERP are required for *L. major* to form infective metacyclics while in the insect foregut (Sádlová *et al*. 2010). However, given their prominent roles at the cell surface, most of these species-specific proteins are likely to have immunological roles. These may be in suppressing innate responses, for example by degrading antimicrobial peptides or other effectors of complement-mediated lysis, as has been shown for PSA (Lincoln *et al.* 2004), or in manipulating cell-mediated immune responses. For instance, TcMUC represses T-cell expansion and cytokine production (Nunes *et al.* 2013). Salivarian trypanosomes employ VSG in antigenic variation, and have evolved sophisticated mechanisms for regulating VSG expression (see below). The abundance and variety of TcMUC, TS and MASP genes has led some to suggest that a subtler form of antigenic variation operates in *T. cruzi* as well (Buscaglia *et al.* 2004, 2006; dos Santos *et al.* 2012).

## GENOMIC INNOVATION: CONTINGENCY ZONES

Trypanosomatids have substantially modified the genome to accommodate these abundant families of cell-surface effectors, by creating genomic sub-domains segregated from the core genome by distance, but also by sequence composition and epigenetic modification (Figueiredo *et al.* 2009; Rudenko, 2010). We can call these sub-domains ‘contingency zones’ because they provide the environment for flexible expression of what are known as contingency genes (Deitsch *et al.* 1997). In this trypanosomatids are not alone; diverse parasites possess polymorphic effector protein families that display specialized expression profiles across a wide range of physiological conditions (Deitsch *et al.* 1997; Kissinger and DeBarry, 2011). It has often been observed that contingency genes aggregate towards the telomeres, a position that promotes both the specific regulation of their expression and their diversification through recombination and gene duplication (Barry *et al.* 2003; Kissinger and DeBarry, 2011). Thus, both *T. brucei* and *T. cruzi* have expanded sub-telomeric regions to contain and regulate their diverse contingency genes (Berriman *et al.* 2005; El-sayed *et al.* 2005*a*, *b*; Moraes Barros *et al.* 2012). It is likely that the strand-switch regions that occur between polycistrons on trypanosomatid chromosomes also serve as incubators of novelty, since they often harbour species-specific genes (Peacock *et al.* 2007; Jackson *et al.* 2009).

Perhaps the best example of structural innovation in trypanosomatid genomes is the VSG expression site (ES) in *T. brucei*. African trypanosomes evade the humoral immune response by periodically switching the VSG monolayer that masks their cell surfaces. This demands that only a single VSG is expressed at a time, while all others are silenced (i.e. monoallelic expression). The function of the ES is to ensure monoallelic expression by providing a dedicated locus for VSG transcription. Thus, the active VSG is transcribed solely from one of several, alternative ESs and antigenic switching occurs when a different VSG from among the many hundreds of silent, sub-telomeric loci, replaces the ES copy through ectopic gene conversion, or by activating an alternative ES (Horn and McCulloch, 2010; Rudenko, 2011). Analysis of ES sequences from several *T. brucei* strains has identified a canonical ES structure (Graham *et al.* 1999; Berriman *et al.* 2002; Hertz-Fowler *et al.* 2008), which includes not only the VSG and repeat sequences required to promote recombination with sub-telomeric VSG loci, but also the ESAGs (reviewed in Pays *et al.* 2001; McCulloch and Horn, 2009). The functions of most ESAGs are unclear; however, all are transcribed preferentially in the bloodstream stage (Jensen *et al.* 2009; Siegel *et al*. 2010; Veitch *et al.* 2010) and it is known that they are *T. brucei-*specific innovations, often derived from conserved gene families with pre-existing cell surface roles (Barker *et al.* 2008; Barnwell *et al.* 2010; Salmon *et al.* 2012; Jackson *et al.* 2013). Hence, it may be that they support antigenic variation or that the specific regulatory environment of the ES has been exploited secondarily to up-regulate proteins with established and diverse roles during the bloodstream stage.

## GENOMIC INNOVATION: THE MAJOR SURFACE PROTEASES

Alongside the many species-specific cell surface proteins, there is one family conserved in all trypansomatid genomes that must have experienced substantial evolution since the origin of parasitism. The Major Surface Protease (MSP) gene family encode a range of metalloproteases that are implicated in various aspects of pathogenesis and virulence in *Leishmania* (Yao, 2010). MSP subverts the normal host defensive mechanisms by degrading components of immune cell signalling pathways (Gomez *et al.* 2009; Hallé *et al*. 2009; Contreras *et al.* 2010), and suppresses other aspects of innate immunity (Kulkarni *et al.* 2006; Lieke *et al.* 2008). In *Trypanosoma*, MSP is equally abundant in gene copy number and protein abundance but its function is less well understood; it is known to remove the VSG coat from the *T. brucei* surface during differentiation into the procyclic form (PCF) (Grandgenett *et al.* 2007) and is thought to have a role in cell invasion by *T. cruzi* (Cuevas *et al.* 2003; Kulkarni *et al.* 2009). As it is present in all trypanosomatids, we can infer the diversification of MSP from its phylogeny, and this too indicates that MSP has been instrumental in parasite adaptation.

**Fig. 3.**
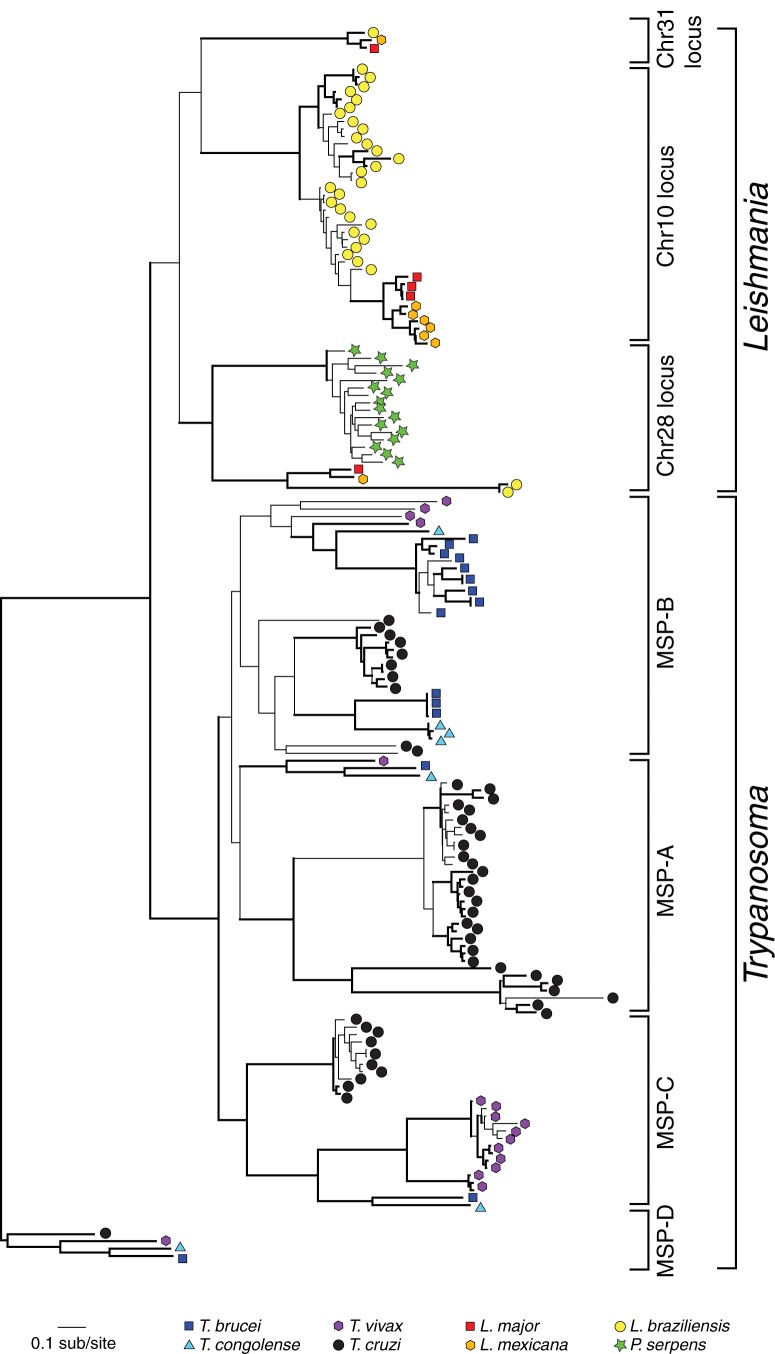
Major Surface Protease (MSP) gene family phylogeny. This maximum likelihood phylogeny of MSP amino acid sequences sampled from completed genome sequences was estimated using PHYML and a LG+Γ model of amino acid substitution (Guindon *et al.* 2010). Node robustness was assessed with non-parametric bootstraps; branches with bootstrap support >75 are shown with bold lines. Sequences are labelled with coloured symbols, according to the key. The tree is mid-point rooted, which corresponds with the *Trypanosoma*-specific MSP-D locus (Marcoux *et al.* 2010).

The MSP phylogeny is described in Fig. 3. It shows how, beginning from a much smaller gene repertoire, MSP has differentiated into distinct clades in both *Leishmania* and *Trypanosoma* (Victoir *et al*. 2005; Ma *et al.* 2011); each clade is associated with a conserved locus, and we know that some of these distinct lineages are developmentally regulated (Yao, 2010). For instance, MSP-A and MSP-C are up-regulated in bloodstream form (BSF) *T. brucei*, while MSP-B is predominantly seen in the procyclic form (LaCount *et al.* 2003; Urbaniak *et al.* 2012). Hence, the trypanosomatids have elaborated their MSP repertoire by creating new loci at least in part to regulate function during the life cycle. Moreover, these different forms have been duplicated to create multiple isoforms, often in species-specific ways; for instance, MSP-C is polymorphic in *Trypanosoma vivax* while single copy in other salivarian species, and the single-copy MSP gene found on chromosome 28 in *Leishmania* has been greatly expanded in *Phytomonas*. However, the phylogeny also demonstrates that MSP in *Leishmania* and *Trypanosoma* cluster by genus, and therefore, there is no orthologous MSP shared by all. Thus, MSP repertoires in *Leishmania* and *Trypanosoma* have evolved independently, and their similarities in genomic structure, developmental regulation and pathogenesis represent parallel evolution, reflecting a common need for diverse surface proteases throughout trypanosomatid diversification.

## DEVELOPMENTAL REGULATION OF GENE EXPRESSION

Trypanosomatids display morphological plasticity that is often associated with developmental transition through a complex life cycle. This is important for the origins of parasitism but not an issue that comparative genomics can illuminate dramatically, without including a comparator lacking developmental complexity. The recent discovery of *Paratrypanosoma confusum* parasitizing the gut of a *Culex pipiens* mosquito strengthens the argument that the ancestral trypanosomatid was a monoxenic insect parasite, since *P. confusum* is a robust outgroup to all other trypanosomatids (Flegontov *et al.* 2013). As long as *P. confusum* has no second host, this shows that a dixenic life cycle has evolved on three separate occasions in *Trypanosoma, Leishmania* and *Phytomonas*. Trypanosomatids are capable of assuming multiple developmental forms and transition between forms coincides with passing between distinct environments, whether they are in different hosts or a single host, for example from the hindgut to the foregut of an insect. Experimental approaches are beginning to reveal the non-coding sequences (Bringaud *et al.* 2007; Holzer *et al.* 2008; Smith *et al.* 2009; Li *et al.* 2012; Pastro *et al.* 2013) and RNA-binding proteins (reviewed in Kolev *et al.* 2014) that interact to regulate gene expression, as well as genes specifically required for differentiation from one life stage to another (Goldenberg and Avila, 2011; Kolev *et al.* 2012; Rico *et al.* 2013). Comparison of life-stage-specific transcriptomes (Holzer *et al.* 2006; Leifso *et al.* 2007; Saxena *et al.* 2007; Rochette *et al.* 2008, 2009; Alcolea *et al.* 2009, 2010; Depledge *et al.* 2009; Jensen *et al.* 2009; Kabani *et al.* 2009; Minning *et al.* 2009; Veitch *et al.* 2010; Adaui *et al.* 2011;) and proteomes (Atwood *et al.* 2005; Rosenzweig *et al.* 2008*a*, *b*; Alcolea *et al.* 2011; Urbaniak *et al.* 2012; Gunasekera *et al.* 2012; Butter *et al.* 2013) in various species have estimated the proportion of genes showing preferential expression in the insect or vertebrate stages to be between 2 and 44%; the breadth of these values reflects the diverse conditions and approaches employed. However, it is clear that a significant minority of genes are developmentally regulated. We can predict that this regulation is achieved with layers of interaction between genomic loci, mRNA, non-coding RNA and DNA and RNA-binding proteins. Hence, to understand the origins of complex life cycles we will need to compare the interaction networks of free-living, monoxenic and dixenic Kinetoplastids, and in this *P. confusum* and the free-living *Bodo saltans* will be instrumental.

## MECHANISMS OF GENOMIC EVOLUTION: GENE DUPLICATION

Besides the genomic innovations themselves, comparative analysis also reveals the molecular mechanisms that create them. These evolutionary events range in size from single amino acid substitutions to chromosomal duplications, and include both coding and non-coding regions, but it is gene duplication above all that creates the raw material for evolutionary novelty (Ohno, 1970). After duplication, paralogs may acquire new functions (neofunctionalization), segregate existing functions (subfunctionalization) or lose function under mutation pressure (pseudogenization) (Lynch and Conery, 2000). Since developmental regulation of gene expression is widespread, it is unsurprising that many gene duplicates are distinguished in the timing or location of their expression. For example, TcMCA5 is an epimastigote-specific metacaspase implicated in programmed cell death of *T. cruzi* that has evolved from a constitutively expressed metacaspase gene family (Kosec *et al.* 2006). In *Leishmania*, Zinoviev *et al.* (2012) identified two functionally redundant RNA helicases that have evolved purely to perform the same role in insect and vertebrate stages respectively. By contrast, TcPRACA and TcPRACB are two paralogous proline racemases involved in immune-suppression by *T. cruzi* (Reina-San-Martín *et al.* 2000); here, function is segregated by location, TcPRACB being expressed intracellularly and TcPRACA secreted (Chamond *et al.* 2005).

Of course, the derivation of many gene duplicates may be multifactorial; in the example of proline racemases, secretion of TcPRACA may coincide with a new role in the differentiation of infective metacyclics (Chamond *et al.* 2005). Thus, it is difficult to unambiguously distinguish neofunctionalization from the segregation of the same function by time, space or substrate. However, the transferrin receptor (TFR) in *T. brucei*, which is required for salvaging haem from the host and is homologous to the VSG (Salmon *et al.* 1997), is one example. Recently, it was confirmed that the TFR had evolved from an a-type VSG in the ancestor of *T. brucei* and *Trypanosoma congolense*, and that, despite their homology, TFR and VSG genes do not recombine, supporting a functionally distinct role from the variant antigen repertoire (Jackson *et al*. 2012, 2013). As suggested above, the conspicuous abundance and diversity of certain *T. cruzi* gene families, such as TS, EF1*γ* and MSP, could indicate that these genes have secondarily evolved a novel role in immune evasion as a consequence of being at the cell surface for their pre-existing functions, i.e. to transfer sialic acid to TcMUC in the case of TS (Oliveira *et al.* 2014). Furthermore, many TS, EF1*γ* and MSP genes in *T. cruzi* are not predicted to encode proteins capable of their putative functions (El-Sayed *et al.* 2005). At first sight, this would appear to indicate frequent pseudogenization, yet a population of pseudogenes acquiring substitutions under neutral conditions would be expected to display a spectrum of mutational decay that is not seen (El-Sayed *et al.* 2005). This suggests that these genes may remain under purifying selection for another role, which could represent neofunctionalization.

**Fig. 4.**
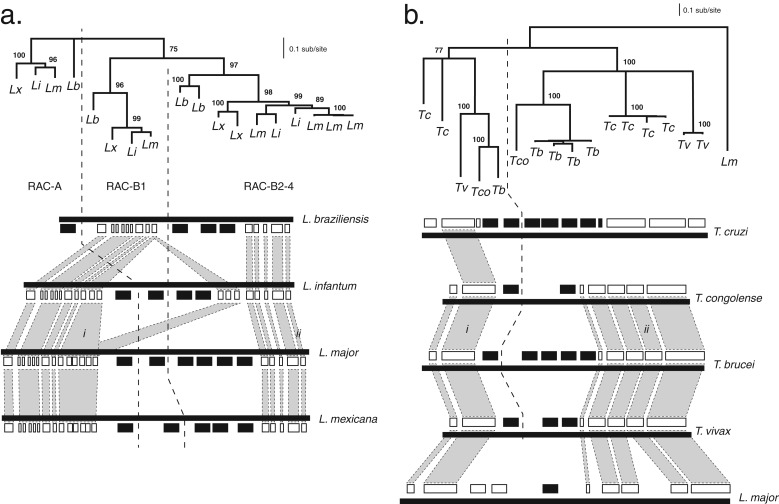
Structural differentiation of paralogues in tandem gene arrays. a. Receptor-type adenylate cyclase (*rac*) genes in four *Leishmania* species. The *rac* array (i.e. LmjF.17·0200) is located at the extreme left-end of chromosome 17 in *L. major*; its conserved position is defined downstream by an EF1*α* gene array (*i*) and upstream by a metalloprotease (*ii*). The structure of the array in four species is depicted, *rac* genes are shown in black, and other loci are shown in white. Vertical grey shading represents homology between flanking loci, to demonstrate positional conservation. The dashed black line separates array positions that correspond with distinct clades in the phylogeny. A maximum likelihood phylogeny estimated from amino acid sequences using a LG+Γ model is shown, with non-parametric bootstraps applied to nodes where support is >75. Terminal nodes are labelled with species name initials. The tree is midpoint rooted. **b**. Cation transporter genes in four *Trypanosoma* species. The transporter array (i.e. Tb927.11.9000) is located on chromosome 11 in *T. brucei*; its conserved position is defined downstream by a palmitoyl acyltransferase 4 gene (*i*) and upstream by an EF1*γ*2 gene (*ii*). The phylogeny and genome comparison are as depicted in **a**. except that the tree is rooted with a single-copy orthology from *L. major*.

The evolution of gene duplicates is particularly obvious in the abundant tandem gene arrays of trypanosomatid genomes. Tandem duplication is very common in trypanosomatids, perhaps as a means of increasing transcript abundance for highly expressed genes in the presence of polycistronic transcription. Comparative analysis of homologous arrays shows that tandem duplicates can evolve new functions, despite the propensity for concerted evolution of tandemly arrayed genes (Jackson, 2007*a*), and that this follows a consistent pattern of structural segregation. Figure 4 shows two examples of functional divergence within tandem gene arrays. The expression profiles of adenylate cyclase gene paralogues from the *rac* array of *Leishmania* spp. correspond with their position in the array. The 3′-most gene (*rac*-A) and the gene positioned upstream of *rac*-A in the array (*rac*-B1) are expressed specifically in the promastigote (Sanchez *et al.* 1995; Akopyants *et al.* 2004), while transcripts for the remaining copies are more abundant in the amastigote (Akopyants *et al.* 2004). Interestingly, *rac*-A and *rac*-B1 may have differentiated in a complementary fashion, since *rac*-B1 negatively regulates the activity of *rac*-A in the promastigote (Sanchez *et al.* 1995). In *Trypanosoma*, the 5′-most copy of a cation transporter gene array is preferentially expressed in the PCF (Jensen *et al.* 2009; Urbaniak *et al.* 2012) (indeed, it is essential to its growth; Alsford *et al.* 2011), while transcripts for all downstream copies are up-regulated in the bloodstream stage (Jensen *et al.* 2009; Veitch *et al.* 2010).

The phylogenies of these gene duplicates show that those gene copies that are functionally differentiated retain orthology across species (i.e. they cluster together despite being in different genomes), while undifferentiated copies cluster by species. This shows that gene duplicates that have diverged in their structures and expression for a novel function are preserved by selection over the course of trypanosomatid evolution, despite the pressure exerted by allelic gene conversion in these situations. In fact, when tandem gene duplicates differentiate, this often occurs at either end of the array (Jackson, 2007*a*), even occurring in otherwise invariant arrays that are exposed to frequent gene conversion; for example, differentiation of the terminal 3′UTR in the *β*-tubulin array in *Leishmania* spp. has created a promastigote-specific *β*-tubulin isoform (Jackson *et al.* 2006).

**Fig. 5.**
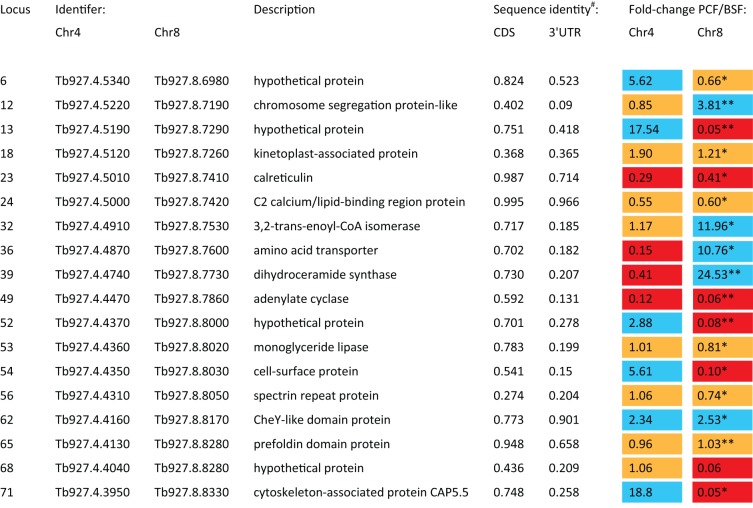
Peptide abundance in procyclic form (PCF) and bloodstream form (BSF) *T. brucei* for selected paralogues resulting from a segmental duplication. 74 loci are present in two forms in *T. brucei* due to a segmental duplication. The loci listed here are those detected in proteomic analyses. Locus number and sequence identity values refer to the segmental duplication described in Jackson (2007*b*). Fold change in peptide abundance between PCF and BSF cells taken from * Urbaniak *et al.* (2012) or ** Butter *et al.* (2013). Preferential expression in PCF and BSF is indicated by blue and red shading respectively. Constitutive expression is indicated by orange shading.

Duplication events do not only affect individual genes. A 0·5 mb segmental duplication in *T. brucei* was identified that has created duplicons shared by chromosomes 4 and 8 (Jackson, 2007*b*). Originally, this region contained approximately 158 genes but subsequent deletions from either duplicon have returned many loci to their original copy number. However, 74 loci have been retained as paralogues in both duplicons. Comparison of their coding and flanking sequences indicated that substantial divergence had occurred and this was assumed to reflect functional divergence (Jackson, 2007*b*). They include CAP5.5, a cysteine peptidase essential for cell morphogenesis, which has been shown to have two paralogues expressed specifically in the insect and vertebrate stages respectively (Hertz-Fowler *et al.* 2001; Olego-Fernandez *et al.* 2009). Figure 5 shows how recent proteomic evidence now confirms that several of the paralogues retained after segmental duplication have evolved stage-specific expression profiles, indicating subfunctionalization by life stage. Gene expression in trypanosomatids is largely regulated by sequences within the 3′ untranslated region (UTR) of transcripts (Vanhamme and Pays, 1995; Haile and Papadopoulou, 2007). Accordingly, it is the paralogous pairs with no sequence identity in their 3′ UTRs that have the greatest differences (loci #13, 36, 39, 49 and 71 in Fig. 5), while those paralogues with similar 3′ UTR sequences display similar abundance in both cases (loci #23, 24, 62 and 65 in Fig. 5).

## MECHANISMS OF GENOMIC EVOLUTION: HORIZONTAL GENE TRANSFER

Horizontal gene transfer (HGT) is another mechanism by which many eukaryotic genomes have acquired new functionality. Berriman *et al.* (2005) identified 49 putative HGT from bacteria and other eukaryotes in trypanosomatid genomes. Confirming HGT rests on sound phylogenetic reconstruction; the most convincing cases are those where the donated gene is closely related to donor genes in unrelated genomes, and nested among these in a phylogeny. Some putative HGT in trypanosomatids achieve this, notably the haem-biosynthesis pathway, absent from *Trypanosoma* but partially restored in *Leishmania* and related genera through HGT of three genes (*hemF, hemG* and *hemH* encoding coproporphyrinogen oxidase, protoporphyrinogen oxidase and ferrochelatase, respectively) from gamma-proteobacteria. In phylogenies, *HemF-H* are nested among bacterial homologues and apart from related eukaryotic genes (Ivens *et al.* 2005, Korený *et al*. 2010). In salivarian trypanosomes, a phospholipase A1 (*PLA1*) gene is thought to have been acquired from proteobacteria (Richmond and Smith, 2007). In support of this, the PLA1 gene is absent from all other Kinetoplastids (indeed most other eukaryotes) and it nests among proteobacterial sequences in sequence comparisons. Moreover, the *PLA*1 locus (Tb927.1.4830) occurs precisely at the boundary between chromosomal core and sub-telomere in African trypanosome genomes, suggesting perhaps that it was recently transposed.

Other good examples of HGT include a cytosolic dihydroorotate dehydrogenase in the pyrimidine biosynthetic pathway, which is unique to Kinetoplastids, and replaces the mitochondrial dihydroorotate dehydrogenase that is typical of euglenids and other eukaryotes. In phylogenies, the cytosolic genes are nested among bacterial taxa, while the mitochondrial genes form a eukaryotic clade (Annoura *et al.* 2005). Likewise, ornithine decarboxylase genes from salivarian trypanosomes do not cluster with homologues from other trypanosomatids, but instead they are nested among metazoan genes and are the sister taxon to ornithine decarboxylase from vertebrates (Steglich and Schaeffer, 2006). In fact, ornithine decarboxylase is known to be absent from *T. cruzi* (Carrillo *et al.* 1999), indicating that this HGT from vertebrates has restored function in African trypanosomes that was lost after the origin of *Trypanosoma*. However, since the African trypanosome genes are not nested *within* the vertebrate clade, we can rule out any recent transfer from contemporary hosts and suggest instead a more distant transfer from an ancient chordate.

In other cases of putative HGT the donated gene is not nested among would-be donors, just closest to them in phylogenies. Here, it is possible that the punctate distribution is due to lineage sorting, i.e. patchy inheritance of an ancestral lineage by daughter lineages. When, as is common, eukaryotic diversity is inadequately sampled, it is difficult to distinguish HGT and lineage sorting. For example, trypanosomatid genomes possess four superoxide dismutase genes required for antioxidant defence (*soda, sodb1, sodb*2 and *sodc*), which localize to distinct cellular compartments (Dufernez *et al.* 2006). The four *sod* genes do not cluster together; *soda/sodc* cluster most closely to *Trichomonas vaginalis*, while *sodb1/sodb*2 cluster with diverse eukaryotes (Dufernez *et al.* 2006). This suggests sorting of ancestral *sod* lineages but not necessarily HGT. Similarly, two metallocarboxypeptidases (TcMCP-1 and TcMCP-2) in *T. cruzi* are found only in Kinetoplastids and prokaryotes, but homologues from the two taxa are sister clades, rather than nested (Niemirowicz *et al.* 2007). While the original study recognized the possibility of both HGT and lineage sorting, they rejected the latter due to the number of deletions this would require. These losses may not be necessary, however, if eukaryotic diversity were exhaustively sampled. Finally, an uncharacterized protein, META1, is up-regulated in *Leishmania* metacyclics and is homologous to a bacterial heat-inducible protein, itself similar to a component of the type III secretion system in *Shigella* (Puri *et al.* 2011). META1 is hypothesized to have evolved via HGT and may be involved in secretory processes in *Leishmania* since mutagenesis of select hydrophobic residues in META1 affects the secretion of the secreted acid phosphatase (Puri *et al.* 2011). However, META1 is not nested among bacterial sequences and, at this stage, the HGT hypothesis rests on it remaining absent from all other eukaryotes.

Although poor sampling continues to limit our ability to distinguish HGT and lineage sorting (Opperdoes and Michels, 2007), HGT has clearly contributed to trypanosomatid genomes; for example, substantial integration of genes from a bacterial endosymbiont has recently been demonstrated in *Angomonas deanei* (Alves *et al.* 2011). The role of HGT in the origins of parasitism will be clarified through comparison of trypanosomatids with free-living Kinetoplastids and other neglected unicellular eukaryotes, to reject the lineage sorting hypothesis and to confirm that the HGT is uniquely associated with parasites, such as *hemF-H* or *PLA1*, and not Kinetoplastids generally.

## CONCLUSION

The genetic content of trypanosomatid genomes indicates that they have been elaborated relative to their common ancestor in terms of both physical structure and physiological capacity. Species-specific gene families, instrumental in cell surface architecture, are central to this history of innovation, and implicitly linked to the origins of complex life cycles and disease. By definition, these unique innovations are mutually exclusive, yet there are themes that cut across species. These gene families are functionally differentiated to perform multiple roles in different host environments through the parasite life cycle. They are positioned in sub-telomeres, tandem gene arrays or other contingency zones that perhaps promote regulatory flexibility and sequence diversity. Their sequences are diverse and often contain low complexity repeats that may promote greater diversity through recombination. In their phylogenies, these gene families display rapid turnover – the gain and loss of lineages – that hint at the importance of host-parasite interactions in genomic evolution. These themes, which would, in fact, apply to parasites of all kinds, suggest how each trypanosomatid lineage has used similar molecular mechanisms to meet the demands of transmission and survival. There are issues in comparative analysis we have not addressed, like protein-protein interactions, the regulatory roles of non-coding regions and regulatory proteins, genomic plasticity or indeed the ~50% of trypanosomatid genes that have no known function. There are also some genes, such as the TcMUC family in *T. cruzi*, procyclin in *T. brucei* and *T. congolense*, and the HASP and SHERP families in *L. major*, that defy any explanation using a comparative approach, and which may have evolved *de novo* from non-coding regions. Yet, we have learned enough from the structure and content of trypanosomatid genomes to conclude that becoming parasitic was more an innovative and elaborative process, than one of loss and reduction. With the addition of free-living Kinetoplastids to our comparative analyses, the mechanisms by which these enigmatic genomic adaptations for parasitism came about will be revealed.
